# Characterization of the complete chloroplast genome of *Zanthoxylum esquirolii* Levl. (Rutaceae)

**DOI:** 10.1080/23802359.2022.2124825

**Published:** 2022-09-23

**Authors:** Xia Liu, Qinqin Huang, Fengting Huang, Chong Sun, Han Liu, Can He, Haowen Liu, Zexiong Chen

**Affiliations:** a College of Horticulture and Landscape Architecture, Southwest University, Chongqing, China; b College of Landscape Architecture and Life Science, Chongqing University of Arts and Sciences, Chongqing, China; c College of Biology and Food Engineering, Chongqing Three Gorges University, Chongqing, China; d College of Horticulture and Gardening, Yangtze University, Jingzhou, China

**Keywords:** *Zanthoxylum esquirolii*, complete chloroplast genome, phylogenetic analysis

## Abstract

*Zanthoxylum esquirolii* Léveillé 1914 is mainly distributed in southwest China, and its wild germplasm resources are scarce and in urgent need of conservation. In this study, we report the first complete chloroplast genome sequence of *Z. esquirolii* using next-generation sequencing. The circular genome is 158,390 bp in length, containing two inverted repeat (IR) regions of 27,622 bp separated by a large single copy (LSC) region of 85,580 bp and a small single copy (SSC) region of 17,566 bp. The chloroplast genome contains a total of 132 genes, including 87 protein-coding genes, 37 tRNA genes, and eight rRNA genes. The overall GC content of the chloroplast genome was 38.46%, with corresponding values in the LSC, SSC, and IR regions of 36.84%, 33.55%, and 42.51%, respectively. The phylogenetic tree revealed that *Z. esquirolii* Levl. formed a clade with *Z. piperitum* DC., *Z. bungeanum* Maxim., *Z. simulans* Hance and *Z. sp.* NH-2018, and had a strongly supported sister relationship with *Z. bungeanum*.

In 1914, Léveillé H. first described of *Zanthoxylum esquirolii* Levl. (Léveillé 1914). *Z. esquirolii* is an important economic forest tree species with good development and utilization value and is an excellent medicinal tree species mainly distributed in the provinces of Guizhou, Sichuan, Tibet and Yunnan in China (Huang, 1997). *Z. esquirolii* has the functions of warming the middle and dispelling cold, promoting blood circulation and relieving pain, and treating cold-related aches and pains, bruises, blood stasis, swelling and pain (He et al., 2011). This plant has great potential for use as a feedstock and medicine. However, the natural habitat of the species’s fragmented, and wild resources of *Z. esquirolii* have been dramatically depleted and need urgent conservation. A large body of knowledge regarding its genetic information would contribute to the formulation of a protection strategy. No genomic information of *Z. esquirolii* has been reported to date. In this study, we present the first complete chloroplast genome sequence of *Z. esquirolii* and construct its phylogenetic relationships with related species based on Illumina paired-end sequencing data. These data will provide a reference for the development and protection of germplasm resources in the future.

The fresh leaves of a single individual of *Z. esquirolii* were collected from Xishui County, Guizhou, China (28.6712° N, 106.4561° E), and a voucher specimen was deposited at the Chongqing University of Arts and Sciences Herbarium (GZLX1) under accession number CUAS-GZ20180720 (Xia Liu, liuxiavip8@163.com). Genomic DNA was extracted using a modified CTAB method (Doyle 1987). The DNA library was sequenced by Hefei Bio&Data Biotechnologies Inc. (Hefei, China) on the BGISEQ-500 platform with PE150 read lengths. The clean reads were used for the de novo assembly of the chloroplast genome using SPAdes Assembler v3.9.0 (Bankevich et al. 2012). With *Z. bungeanum* Maxim. (NC_031386) as the reference. The annotation of the complete genome was performed using CpGAVAS (Liu et al. 2012) and GeSeq software (Michael et al. 2017). After a manual check and adjustment, the annotated chloroplast genome sequence of *Z. esquirolii* was submitted to GenBank (MZ676709).

The chloroplast genome of *Z. esquirolii* is a double stranded, circular DNA 158,390 bp in length that contains two inverted repeat (IR) regions of 27,622 bp separated by a large single-copy (LSC) region and a small single-copy (SSC) region of 85,580 bp and 17,566 bp, respectively. The chloroplast genome encodes a total of 132 genes (87 protein-coding, 37 tRNA, and 8 rRNA genes), with 18 duplicated genes (7 protein-coding, 7 tRNA, and 4 rRNA genes). Nineteen genes contain two exons and four protein-coding genes (*ycf3*, *clpP*, and two *rps12*) contain three exons. The overall GC content of *Z. esquirolii* is 38.46% and the values in the LSC, SSC and IR regions are 36.84%, 33.55%, and 42.51%, respectively.

The phylogenetic analysis was performed using 12 complete plastid genomes, with *Phellodendron amurense* Rupr. and *Phellodendron chinense* Schneid. as the outgroup (Figure 1). The 12 complete chloroplast genome sequences were subjected to multiple sequence alignment using MAFFT software (Katoh and Standley 2013). The best models of the complete chloroplast genomes for the ML and BI phylogenetic analyses were determined by jModelTest 2.1.1 (Posada 2008) with an Akaike Information Criterion (AICc). The best fitting evolutionary model for the combined complete chloroplast genome dataset was TVM + I + G in the ML and Bayesian analyses. A maximum likelihood (ML) phylogenetic tree was built using the RAxML version 8 program (Alexandros 2014) with 1000 bootstrap replicates. For BI in MrBayes 3.1.2 (Ronquist and Huelsenbeck 2003), two independent Markov chain Monte Carlo (MCMC) runs were performed and contained four MCMC chains that were run for 1000,000 generations and sampled every 1000 generations; all other parameters were set to default. The first 25% of the sampled trees were abandoned as burn-in to check the stability of each run, and the posterior probabilities (PP) were calculated from the remaining trees. All phylogenetic trees were viewed using the Figtree v1.4.2 program (Rambaut 2014). The phylogenetic trees generated by the ML and Bayesian methods were most similar to each other but with different branch support values in some clades (Figure 1). Phylogenetic analysis showed that the *Zanthoxylum* species formed a monophyletic group. *Z. esquirolii* is most closely related to *Z. bungeanum* and is sister to *Z. sp.* NH-2018 and *Z. simulans* Hance, with 100% bootstrap support.

**Figure 1.**
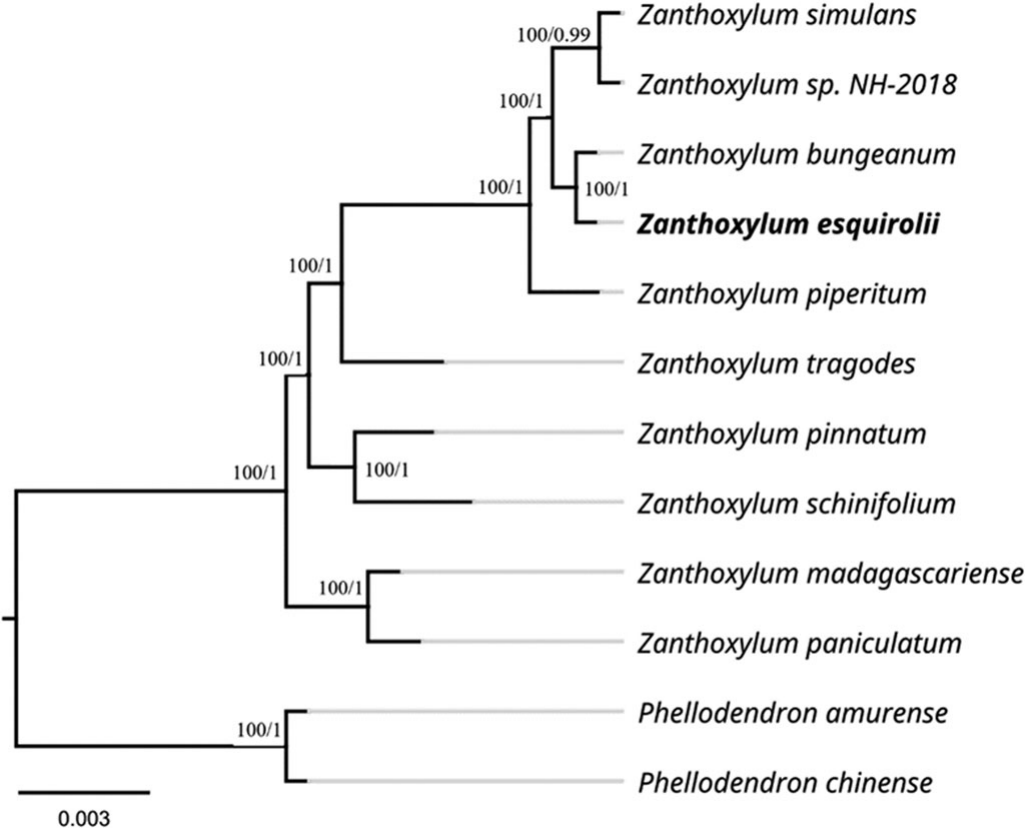
Maximum-likelihood phylogenetic tree of *Z. esquirolii* and other related species based on complete chloroplast genome sequences. Numbers near the nodes sequentially indicate ML/BI support values. The following sequences were used: *Zanthoxylum simulans* Hance NC_037482 (Hou et al. 2017), *Zanthoxylum sp.* NH-2018 MF716521, *Zanthoxylum bungeanum* Maxim NC_031386 (Liu and Wei 2017), *Zanthoxylum esquirolii* Levl. MZ676709, *Zanthoxylum piperitum* Maxim NC_027939 (Lee et al. 2016), *Zanthoxylum tragodes* NC_046747, *Zanthoxylum pinnatum* NC_046746, *Zanthoxylum schinifolium* Sieb. et Zucc. NC_046746, *Zanthoxylum madagascariense* NC_046744, *Zanthoxylum paniculatum* NC_046745, *Phellodendron amurense* Rupr. NC_035551 and *Phellodendron chinense* Schneid. MT916287.

The limited number of polymorphic loci produced by low-resolution markers hinders the phylogenetic research on *Zanthoxylum* species in previous studies (Medhi et al. 2014; Feng et al. 2015; Kim et al. 2017; Appelhans et al. 2018). The emergence of the chloroplast genomes of a large number of *Zanthoxylum* species can provide important insights into the evolution of *Zanthoxylum* in eastern Asia. This complete chloroplast genome can be used for phylogenetic, population, and chloroplast genetic engineering studies of *Z. esquirolii* and is fundamental for the creation of new conservation and management strategies for this important medicinal plant species.

## Data Availability

The genome sequence data that support the findings of this study are openly available in GenBank of NCBI at (https://www.ncbi.nlm.nih.gov/) under the accession no. MZ676709. The associated BioProject, SRA, and Bio-Sample numbers are PRJNA680256, SRR17163937, and SAMN23766595, respectively.
